# The prognostic significance of serum gamma-glutamyltransferase levels and AST/ALT in primary hepatic carcinoma

**DOI:** 10.1186/s12885-019-6011-8

**Published:** 2019-08-27

**Authors:** Li-xiang Zhang, Yang Lv, A-Man Xu, Huan-zhong Wang

**Affiliations:** 1 Department of Gastrointestinal Surgery, the First Affiliated Hospital of Anhui Medical University, Hefei, China; 2 Second People’s Hospital of Jingmen City, Hubei Province, China; 3 the tenth oncology department, Hefei Cancer Hospital of Chinese Academy of Sciences, Hefei, China

**Keywords:** Gamma-glutamyltransferase, Aminotransferase, Aspartate aminotransferase, Prognosis

## Abstract

**Background:**

Blood counting and the liver function tests, as the routine examinations, can reflect the immune and nutritional status of the body, our aim is to assess the prognostic significance of serum gamma-glutamyltransferase (GGT) levels and AST/ALT in primary hepatic carcinoma.

**Methods:**

Clinico-pathological data of 414 patients with primary hepatic carcinoma in the 1st Affiliated Hospital of Anhui Medical College between January 2007 to January 2014 was analyzed retrospectively in this study. Survival curves were described by Kaplan-Meier method and compared by Log-rank test, univariate and multivariate analysis were used to identify the prognostic factors.

**Results:**

GGT was positively correlated with the tumor size(*P* = 0.000), tumor volume (*P* = 0.000), tumor volume percent (*P* = 0.004), TNM stage(*P* = 0.009), 1-year survival rate (*P* = 0.000), 3- years survival rate (*P* = 0.000) and 5-years survival rate(*P* = 0.000). The serum ALT/AST was significantly correlated with age (*P* = 0.047), tumor size(*P* = 0.002), tumor volume (*P* = 0.010), tumor volume percent (*P* = 0.005), TNM stage(*P* = 0.006), liver cirrhosis(*P* = 0.003), 3- years survival rate (*P* = 0.032) and 5-years survival rate(*P* = 0.000). The Kaplan-Meier curves showed that the patients with primary hepatic carcinoma had a longer time in the low GGT group and low AST/ALT group, showing a significant difference (*P* < 0.05). The univariate and multivariate analyses showed that TNM stage, differentiation grade, tumor volume, GGT and AST/ALT were independent factors for predicting overall survival rate of primary hepatic carcinoma patients.

**Conclusions:**

GGT and AST/ALT were independent factors for predicting overall survival rate of primary hepatic carcinoma patients.

## Background

Hepatocellular carcinoma (HCC) is the six cause of tumor-related mortalities worldwide and refers to the most general kind of primary liver tumor [1, 2]. Hepatectomy is the main treatment for HCC. Although the prognosis of HCC has improved with the improvement of surgery approaches, the five-year surviving ratio of the patients having advanced HCC or metastatic HCC is still very low [3, 4]. The prognosis of HCC is closely related to early finding and early therapy. At present, the main diagnostic methods of hepatocellular carcinoma include abdominal ultrasonography, CT (namely computer tomography), MRI (namely the magnetism resonance image) and liver biopsy. Because of the high cost of these imaging techniques, invasiveness and trauma of liver biopsy, clinical studies have begun to explore simple prognostic blood markers.

In the clinical, complete blood counting and liver function tests (containing NLR, the abbreviation of the neutrophil-lymphocyte rate [5, 6], PLR, the abbreviation of platelet-lymphocyte rate [7, 8], ALT, the abbreviation of alanine aminotransferase, AST, the abbreviation of aspartate aminotransferase [9, 10], GGT, the abbreviation of gamma glutamyltransferase [11, 12]), which can reflect the immune and nutritional status of the body, were reported to be the predictors of OS in some tumors. Among them, PLR and NLR as predictors have been studied worldwide in recent years. Additionally, ALT, AST and GGT [13] may be related with the survival of cancer patients. Although ALT, AST and GGT have certain reference value in the diagnosis of HCC compared with AFP, these indexes also play important role in the liver. γ-glutamyltransferase (GGT) is a key enzyme in the process of biotransformation and nucleic acid metabolism, recent study show that it can also lead to tumorigenesis and characterized as a marker for HCC [14]. Aspartate aminotransferase /alanine aminotransferase (AST/ALT) also has important clinical significance in the prognosis of some cancer [15, 16]. However, the prognostic value of AST/ALT and GGT have not been explored deeply and widely in HCC patients after hepatectomy. Within this research, our group explore the prognostic value of AST/ALT and GGT and the relationship between AST/ALT and GGT with clinicopathological parameters. Besides, we also compared the prognostic value of other blood indices and models with HCC.

## Methods

### Ethics

The research got approval from the Ethics Committee in the 1st Affiliated Hospital of the Anhui Medical College. Informed consent in the written form was gained from the patients. This research got carried out according to the guidance from Statement of Helsink.

### Patients

Clinico-pathological statistics of 414 patients, who were examined as HCC and received curative hepatectomy during the 1st Affiliated Hospital of Anhui Medical College from January 2007 to January 2014, were analyzed retrospectively during the research, we gathered their basic information through the medical record room of our hospital. Information were gathered regarding fundamental characteristics of patients (Table 1), containing their blood examinations (neutrophil, lymphocyte, platelet, alanine aminotransferase (namely ALT), the aspartate transaminase (namely AST), theγ-glutamyl transpeptidase (namely GGT), and alpha-fetoprotein (AFP) and pathological results (Child-Pugh classification, Tumor Node Metastasis (TNM) staging system, tumor size, differentiation grade, tumor volume, tumor volume percent, vascular invasion, nerve invasion). Microscopic observation was used to determine whether nerves and vessel are invaded by tumors. The disease progression within the HCC patients got divided applying the guidance summarized in the eighth vision of the AJCC, namely the American Joint Conference upon tumor concerning TNM (tumor-node-metastasis) staging.

**Table 1:** Characteristics of the recruited patients

Characteristics	Median(25th–75th percentile) or no. (%)
Gender	
male	332 (80.2)
female	82 (19.8)
Age (year)	
< 60	246 (59.6)
≥ 60	168 (40.4)
Tumor size	
< 5 cm	286 (69.1)
≥ 5 cm	128 (30.9)
TNM stage	
I- II	361 (87.2)
III-IV	53 (12.8)
Differentiation grade	
low	45 (10.9)
moderate	268 (64.7)
high	101 (24.4)
Liver cirrhosis	
yes	251 (60.6)
no	163 (39.4)
5-year survival	
yes	211 (51.0)
no	203 (49.0)
Tumor volume (cm^3^)	33.49 (8.18–179.50)
Tumor-volume percent	0.0338 (0.0081–0.1521)
AFP	20.15 (4.7–339.2)
AST	39(29.00-59.00)
ALT	37(26.00-55.00)
GGT	98.18 (35.00–112.00)
Neutrophil count	3.8029 (2.4000–4.2000)×10^9^/L
Platelet count	143.826 (93.0000–181.25000)×10^9^/L
Lymphocyte count	1.4522 (1.000–1.8000)×10^9^/L

### Definition of prognostic factors

Peripheral blood tests were obtained within 1 week before surgery, we determine the following indexes (NLR, PLR, and tumor volume percent). NLR got counted with the means of dividing the strict neutrophil counting with the strict lymphocyte counting, PLR was counted by dividing the strict platelet count by the strict lymphocyte count, tumor volume percent was got by dividing the tumor volume by the whole liver volume. The recommended cutoff value for preoperative PLR, NLR, AST/ALT and GGT were decided by applying ROC (recipient operation characteristics) curve on the basis of the Youden finger [maximum (sensitivity+ specificity-1)] [17].

#### Inclusion and exclusion criteria

The eligibility criteria included: 1) All patients were confirmed HCC by pathological diagnosis; 2) Child-Pugh grade A or B; 3) these patients didn’t have heart sickness or any importan organs failures; 4) the need of surgery was definite; 5) their peripheral blood tests were obtained within one week before surgery.

Excluding standards from this research contained the situations as follows; 1) they had previous malignant tumors or various primary tumors; 2) they had accepted radiation treatment or chemo treatment previously before the treatment; 3) they had certain disease that could influences the counting of peripheral blood cells, such as infection; 4) they passed away within thirty days after the operation during the period of the follow-up.

### Follow-up and treatment

This research gathers 488 examples of HCC patients having complete information. After surgery, some patients accepted local treatment, including ablation and transarterial embolization, some patients accepted chemotherapy or targeted therapy, patients in poor health received supportive treatment. Except cirrhosis, chronic hepatitis or other liver dysfunction occurs in patients, we assessed their Child Classification based on their liver function. Their follow-up date were obtained through telephones and outpatient visit. Enrolled patients got prospective follow-up. This behavior got carried out in normal intervals (each 90 days within two years after the surgery, each 180 days within the years of three-five, and once a year after five years). We followed up all the patients, 74 patients got exclusion from this research, among them, 54 lost contact, 12 died of non-cancer-related deaths and 8 died within 30 days after surgery, in the end, 414 HCC cases got included in the final analysis of this study.

### Statistical explanation

The whole data explanations got carried out applying SPSS app (16.0 version). The cutoff value of some serum indexes was performed according to the ROC curve. The connections between AST/ALT and GGT levels with clinico-pathological features were measured by the χ2 examinations or the Fisher accurate examination, as appropriate. For analysis of overall surviving, Kaplan-Meier curves got established using the log-rank test. The multivariate and univariate surviving analysis were carried out using the Cox appropriate hazard pattern. For the whole examinations, *P* < 0.05 got thought to be statistically significant.

## Results

### Patient features

The patients features are expressed in the Table 1, Overall, 332 (83.6%) patients turned out to be males and 82 (24.2%) were females. The median age of the patients was 56 (scope, 25–82). The median following-up month was 56.4 (scope, 1–103 months). A tumor ≥5 cm got observed in 128 (30.9%) patients, the median tumor volume was 204.26cm^3^(8.18–179.50), the median tumor volume percent was 0.0338(0.0081–0.1521), the pathological stage of I-II and III-IV got observed in 361 (87.2%) and 53 (12.8%) patients, respectively. The liver cirrhosis of patients were 251, other patients have chronic hepatitis or other liver dysfunction, such as alcoholic liver disease and HBV infected liver disease. The differentiation grade was divided into poor grade (45), moderate grade (268) and high grade (101). During the follow-up period, the 12-, 36-, and 60-month surviving ratios turned out to be 87.4, 69.6 and 50.5%, respectively. In the end of this five-year following-up process, 203 (49%) patients had passed away, 282 patients had recurrence of hepatocellular carcinoma within 5 years after operation, the recurrence rate was 68.1%, the recurrence-free survival rate was 31.9%. And the median follow up data was 56.4 months.

### Cutoff values of prognostic factors

**Fig. 1:**
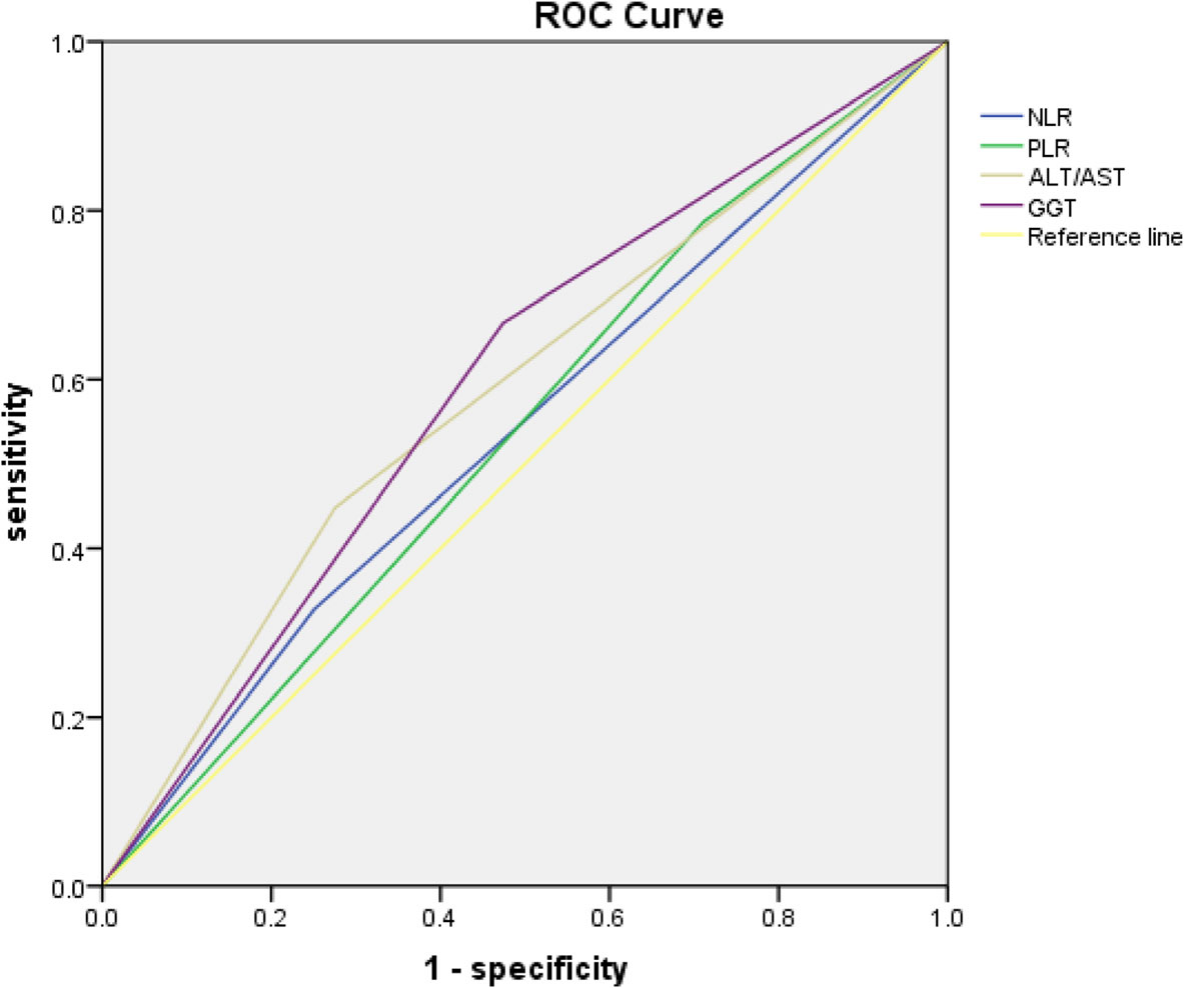
ROC curves for serum makers in patients

Using the ROC curve, we decided that the recommended cutoff values of NLR, PLR, AST/ALT and GGT were 3.11 [sensitivity, 32.5; specificity, 74.9; AUC (area under the ROC curve), 0.539; *P* = 0.004], 70.79 g/L [sensitivity, 78.6; specificity, 28.9; AUC, 0.537; *P* = 0.037], 1.26 [sensitivity, 44.8; specificity, 72.5; AUC, 0.588; *P* = 0.002] and 48.5[sensitivity, 67.0; specificity, 52.6; AUC, 0.606; *P* = 0.000], respectively (Fig. 1).

### Relationship of baseline GGT levels and ALT/AST with clinico-pathologic features

The connection *was displayed in* Table 2*. The serum GGT wasn’t significantly correlated with age, gender, nerve invasion, vascular invasion, Liver cirrhosis or differentiation grade, however, the association between GGT and tumor size(P = 0.000), tumor volume (P = 0.000), t*umor volume percent (*P* = 0.004), TNM stage(*P* = 0.009), 1 year survival rate (*P* = 0.000), 3 years survival rate (*P* = 0.000) and 5 years surviving rate(*P* = 0.000) were significant.

**Table 2:** Relationship between the GGT and AST/ALT with clinicopathologic characteristics

Patient-related factors	GGT (mg/L)		*P* value	AST/ALT		*P* value
	< 48.5 (*n* = 178)	≥48.5 (*n* = 236)		< 1.26 (*n* = 265)	≥1.26 (*n* = 149)	
Gender			0.351			0.249
male	139	193		217	115	
female	39	43		48	34	
Age (years)			0.062			0.047*
< 60	115	131		167	79	
≥ 60	63	105		98	70	
Tumor sizes			0.000*			0.002*
< 5 cm	142	144		197	89	
≥ 5 cm	36	92		68	60	
Tumor volume			0.000*			0.010*
< 33.49	95	84		127	52	
≥ 33.49	83	152		138	97	
Tumor volume percent			0.004*			0.005*
< 0.5	169	204		247	126	
≥ 0.5	9	32		18	23	
TNM stage			0.009*			0.006*
I,II	164	197		240	121	
III,IV	14	39		25	28	
Vascular invasion			0.052			0.978
yes	14	33		30	17	
no	164	203		235	132	
Nerve invasion			0.131			0.267
yes	0	3		1	2	
no	178	233		264	147	
Differentiation grade			0.896			0.193
well	43	58		26	19	
moderate	117	151		180	88	
Poor	18	27		26	19	
Liver cirrhosis			0.318			0.003*
yes	103	148		175	76	
no	75	88		90	73	
Death(1 year)			0.000*			0.102
yes	7	45		28	24	
no	171	191		237	125	
Death(3 year)			0.000*			0.032*
yes	33	93		71	55	
no	145	143		194	94	
Death(5 year)			0.000*			0.000*
yes	67	136		112	91	
no	111	100		153	58	

The * was considered to be statistically significant

The serum ALT/AST was significantly connected with age (*P* = 0.047), tumor size(*P* = 0.002), tumor volume (*P* = 0.010), tumor volume percent (*P* = 0.005), TNM stage(*P* = 0.006), liver cirrhosis(*P* = 0.003), 3 years survival rate (*P* = 0.032) and 5 years survival rate(*P* = 0.000), there were no relationship between ALT/AST with gender, nerve invasion, vascular invasion, one year survival rate or differentiation grade.

### The Kaplan-Meier survival curves of patients

**Fig. 2:**
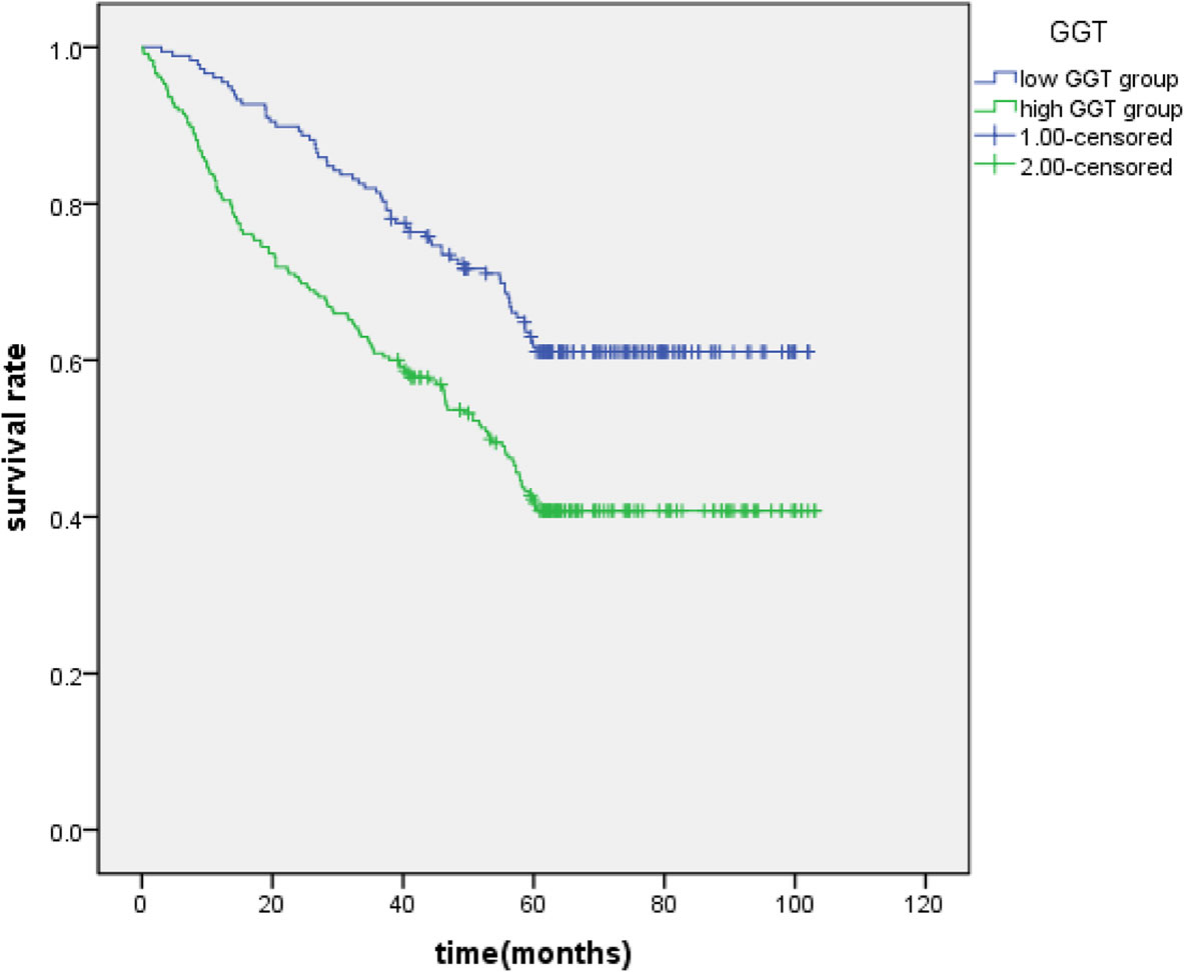
Kaplan-Meier curves of GGT

**Fig. 3:**
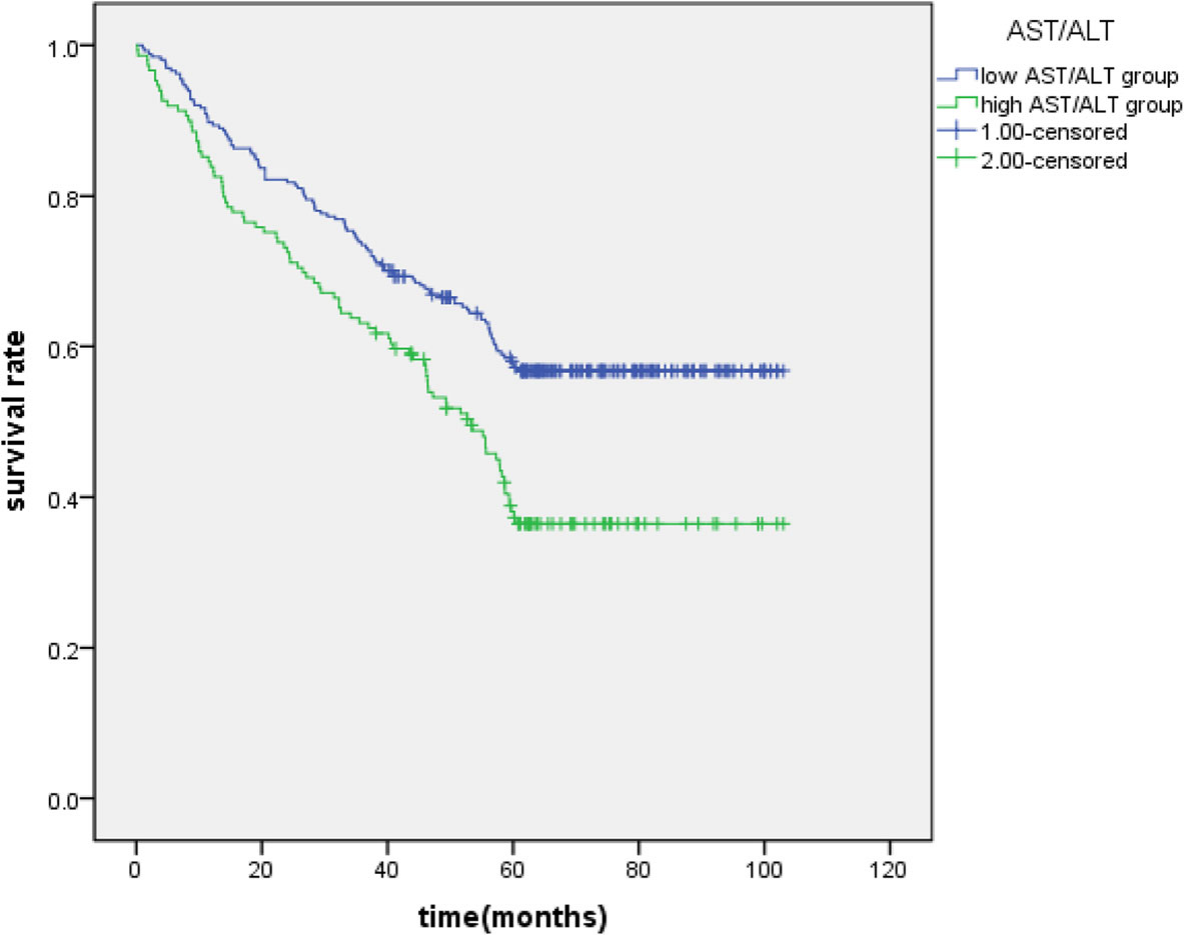
Kaplan-Meier curves of AST/ALT

**Fig. 4:**
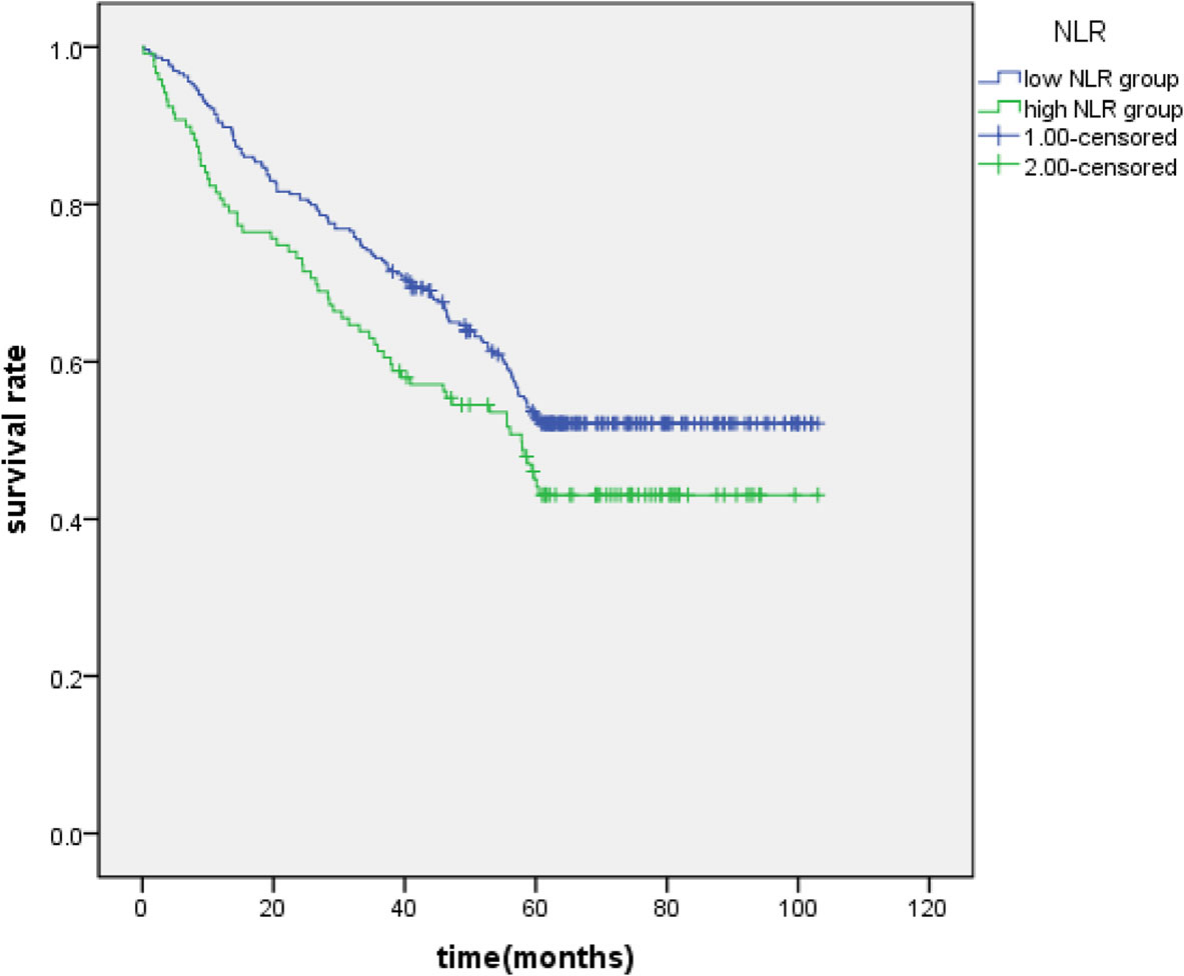
Kaplan-Meier curves of NLR

The Kaplan-Meier survival curves for patients in high GGT and low GGT group (Fig. 2), high AST/ALT and low AST/ALT (Fig. 3), high NLR group and low NLR group(Fig. 4) were expressed. The patients with GGT < 48.5 (*P* = 0.000), AST/ALT< 1.26 (*P* = 0.000), NLR < 3.11 (*P* = 0.043) had longer overall surviving .

### The risk factors for overall surviving

As indicated by univariate analysis, tumor size, TNM stage, differentiation grade, tumor volume, tumor volume percent, vascular invasion, GGT, AST/ALT and NLR importantly influenced the overall surviving (Table 3). And multivariate analysis indicated that TNM stage, differentiation grade, tumor volume, GGT and AST/ALT were independent risk factors.

**Table 3:** Univariate and Multivariate analyses of factors for prediction of overall survival

Characteristics	Univariate analysis		Multivariate analysis	
	*HR value (95% CI)*	*P* value	*HR value (95% CI)*	*P* value
Gender (male / female)	0.498			
Age(<60 years / ≥60 years)	0.926(0.699,1.226)	0.590		
Tumor size (<5 cm / ≥5 cm)	2.095(1.582,2.77)	0.000	1.232(0.866,1.753)	0.245
TNM stage ( I, II / III,IV )	3.393(2.428,4.741)	0.000	2.482(1.717,3.587)	0.000
Differentiation grade ( well /moderate/ poor)	0.628(0.489, 0.806)	0.000	0.670(0.524, 0.855)	0.001
Tumor volume	1.746(1.308,2.332)	0.000	1.221(1.054,1.415)	0.008
Tumor volume percent	2.524(1.727,3.687)	0.000	1.289(0.796,2.088)	0.302
Vascular invasion	1.661(1.121,2.462)	0.011	1.089(0.707,1.678)	0.699
Nerve invasion	1.434(0.356,5.775)	0.612		
Liver cirrhosis	0.875(0.660, 1.161)	0.355		
GGT	1.930(1.440,2.588)	0.000	1.706(1.260,2.311)	0.001
ALT/AST	1.698(1.286,2.241)	0.000	1.424(1.064,1.905)	0.017
AFP	1.303(0.940,1.806)	0.112		
NLR	1.352(1.008,1.814)	0.044	1.888 (0.872,1.619)	0.275
PLR	1.337(0.954,1.873)	0.092		

## Discussion

Hepatectomy is the most important treatment for HCC worldwide. Due to the limitation of diagnostic techniques, it is often difficult to detect early HCC and lead to poor prognosis. At present, 5 years of postoperative recurrence rate is high [18]. Therefore, in order to improve the prognosis of HCC, many scholars have made a lot of contributions. Studies have shown that elevated levels of markers may be associated with prognosis of HCC patients. Lymph node metastasis, tumor size, differentiation grade, TNM stage and vascular invasion were defined as prognostic factors. However, these prognostic factors are difficult to judge before surgery, so the research on prognostic serum markers has been explored widely in recent years.

γ-glutamyltransferase (GGT), as an independent prognostic indicator of tumor-related diseases, has been concerned by more and more researchers. GGT was related with the prognosis of renal cell carcinoma, ovarian cancer, endometrial carcinoma, and esophageal squamous cell carcinoma [19–21]. In this paper, we found that patients with preoperative GGT ≥ 38.5 u/L had aggressive liver disease and shorter overall survival *(P* < 0.05), indicating that GGT was significantly correlated with the pathological results and prognosis of primary liver cancer patients.

The mechanism of γ-GT and tumor progression remains unclear. GGT is a membrane-bound enzyme involved in the metabolism of glutathione (GSH) by transferring γ-glutamyl, and GSH has been identified antioxidant in the cell, it can protects cells from oxidant damage by neutralizing reactive oxygen species and free radicals [22, 23]. Therefore, GGT levels will increase in the pathological state of oxidative stress. However, when the expression of GGT is too high, it can disturb the balance of oxidant and anti-oxidation, leading to sustained oxidative stress in tumor cells, which can contribute the process of cancers [24]. Apart from being a diagnostic marker for hepatobiliary disease, GGT also regulates cell proliferation and apoptosis, and play important roles in cancer progression, invasion, and resistance to anticancer drugs [25].

In this study, we could concluded that the association between GGT and tumor size(*P* = 0.000), tumor volume (*P* = 0.000), tumor volume percent (*P* = 0.004), TNM stage(*P* = 0.009), 1 year survival rate (*P* = 0.000), 3 years survival rate (*P* = 0.000) and 5 years survival rate(*P* = 0.000) were significant, GGT may play an important role to help evaluate the prognosis and progression of liver cancer patients.

AST (aspartate aminotransferase) and ALT (Alanine aminotransferase) are also significant enzymes in the liver [26]. ALT is mainly found in non-mitochondria of hepatocytes, while AST is mostly present in mitochondria of hepatocytes. Advanced liver disease is associated with mitochondrial damage, thus this can directly release AST into the bloodstream leading to a dramatic increase in serum levels. On the other hand, as the liver function decrease, the clearance rate of AST also decreases [27]. Therefore, serum AST level is significantly higher than serum ALT level. In the research, patients with higher AST/ALT level had a poor prognosis than patients with lower AST/ALT level, and AST/ALT was a risk factor of overall survival with primary liver cancer patients. The level of AST/ALT ratio is also closely related to residual hepatic inflammatory necrosis [28], our study was consistent with this, the high AST/ALT level of patients can reflect severe liver necrosis, which leads to the invasion and recurrence of hepatocellular carcinoma. But the mechanism of ALT/AST level connecting with the prognosis of HCC patients needs further research.

Tumor volume, differentiation grade, TNM stage, GGT and AST/ALT levels were independent risk factors for the prognosis of the patients with primary liver cancer (*P* < 0.05), indicating that GGT and AST/ALT also has the significant predictive value for primary liver cancer. TNM stage and tumor volume, differentiation grade are difficult to diagnose by imaging methods before surgery, but preoperative GGT and ASL/ALT are easier to obtain and can provide some guidance for treatment. Early treatment can be performed on patients with high preoperative GGT and AST/ALT level, for example, we can use vascular embolization to reduce the progression of tumor invasion before surgery to improve the prognosis of these patients, this may help prevent dangerous situations such as recurrence and metastasis. However, we can not only use the GGT level to judge the prognosis, the GGT, AST/ALT, TNM stage, ascites, cirrhosis and other indicators should also be considered comprehensively. Adequate evaluation and surgical preparations are needed before surgery to make the best treatment plan for different patients, after surgery, follow-up and observation are worthy noted to detect early recurrence or metastasis to improve the survival time of patients.

## Conclusions

The patients having primary hepatic carcinoma had a longer time in the low GGT group and low AST/ALT group, besides, TNM stage, differentiation grade, tumor volume, GGT and AST/ALT were independent factors for predicting overall survival rate of primary hepatic carcinoma patients.

## Data Availability

Due to ethical restrictions, the raw data underlying this paper are available. upon request to the corresponding author.

## Abbreviations

ALT: Aminotransferase
AST: Aspartate aminotransferase
GGT: Gamma-glutamyltransferase
NLR: Neutrophils to lymphocytes ratio
PLR: Platelets to lymphocytes ratio
